# Opposing Effects of Additives in Dry Milling and Tableting of Organic Particles

**DOI:** 10.3390/pharmaceutics13091434

**Published:** 2021-09-09

**Authors:** Lina Miethke, Paul Prziwara, Jan Henrik Finke, Sandra Breitung-Faes

**Affiliations:** 1 Institute for Particle Technology, Technische Universität Braunschweig, 38104 Braunschweig, Germany; l.miethke@gmx.de (L.M.); p.prziwara@tu-braunschweig.de (P.P.); 2 Center of Pharmaceutical Engineering—PVZ, Technische Universität Braunschweig, 38106 Braunschweig, Germany

**Keywords:** organic particles, additives, dry milling, tableting, compaction, lubricants, flow aids, grinding aids

## Abstract

Applying additives and excipients during the dry processing of fine particles is a common measure to control the particle–particle interactions, to specifically influence the powder properties and to enhance the process efficiency or product quality. In this study, the impacts of a particulate lubricant, a nano-disperse flow additive and liquid grinding aids on the dry fine milling and subsequent tableting of the ground material were investigated for three different organic model compounds. It is presented that the three additive classes cause varying and partly opposing effects during these process steps. Especially the lubricant and the grinding aids were shown to increase the efficiency of the milling process as well as the product fineness of the ground material, and to avoid critical product adhesions on the machine surfaces. Thereby, stable and efficient grinding conditions were partially not possible without the addition of such additives. However, as these positive effects are attributed to a reduction of the adhesive forces between the particles, much lower tablet strengths were achieved for these additives. This propagation of powder, and in turn, final product properties over whole process chains, has not been studied in detail so far. It was further revealed that the material behavior and the microstructure of the product particles is decisive for the processing as well, which is why additive effects may be product-specific and can even be suppressed under certain processing conditions. In comparison to the process performances, the powder properties and surface energies of the product particles were less influenced by the additives. On the contrary, particle-based morphologies or deformation behavior seem to play a major role in comparison to inorganic materials. Thus, it can be stated that global bulk properties and surface energies provide first indications of powder behavior and susceptibility. However, additional specific properties need to be evaluated to more clearly understand the influences of additives.

## 1. Introduction

Fine organic particles play an important role in many different industries, such as the (agro) chemical and pharmaceutical industry, the field of pigments and cosmetics or food production. Since many organic materials show insufficient stability in a wet state or the subsequent drying process would consume too much energy, the processing of these particles is often performed in dry mode. Thereby, different unit operations such as grinding, classification, blending, conveying, filling, dosing or compacting are usually combined along the process chain to transfer the particles into the final product. Currently, an increasing demand for fine particles is seen in many industries due to various reasons. For instance, poorly soluble drugs with high specific surface areas often show an improved bioavailability [1,2], organic pigments may have enhanced color strengths above a certain fineness [3] or micronized polymers with narrow size distribution can optimize additive manufacturing processes [4]. In this regard, fine grinding is a common option to achieve the required product qualities by increasing the specific surface area and/or adjusting the particle size distribution. For industrial dry fine grinding to the lower micron range, jet mills are currently the most appropriate option, since they enable the production of fine particles at relatively high production capacities (see, e.g., [5,6]).

Generally, the applied unit operations are highly sensitive towards the flow and bulk behavior of the particles. Especially for fine products, where the powder properties are strongly determined by the particle–particle interactions, the powders are often difficult to control and to handle. This is the reason why different kinds of additives can be applied along the process chain, depending on the unit operation, the material-specific product behavior and their general applicability in the desired field. In the field of organic materials, mostly solid additives are added to control the bulk and powder behavior, e.g., flow additives and lubricants, which are coated on the solid surface of the host particles by means of different kinds of impact or high-shear blenders [7,8]. Flow additives usually consist of nanoscale particles, which keep their host particles out of the range of highly attractive forces [9,10]. Lubricants are mostly applied during tableting. They primarily act by reducing the frictional forces during tableting, and thus, by preventing particle adhesion on the punch surfaces and by reducing friction at the machine surfaces, but sometimes also by improving the powder flow [11,12,13].

Liquid grinding aids constitute another important additive class, especially in the field of dry fine grinding of inorganic materials. The additive molecules reduce the surface energy of the solid surface by adsorbing on the particle surface and prevent direct contact between the particles, which leads to a lower tendency of agglomeration and an improved powder flow behavior [14,15,16]. Besides improving the final powder properties, the major aims of applying these additives are therefore to (a) increase the production capacities, (b) reduce the energy consumption or (c) enable the production of even finer particles [17]. These additives are mainly applied for dry fine grinding in mills, such as tumbling ball mills [18,19,20,21,22] or stirred media mills [23,24,25], and vertical roller mills [26]. Moreover, a few studies reveal high potential for applying grinding aids in fluidized bed-opposed jet mills [27,28,29]. The understanding of grinding aids in jet mills is still comparatively marginal. Regarding the application of grinding aids in fine grinding, organic materials have hardly/barely been studied so far. Only Fukumori et al. [30] showed that these additives principally also work for organic materials such as chitosan powder. The low number of studies is even more remarkable, as many organic materials are known to form critical material adhesion on the mill surfaces due to agglomeration [6], which can normally be effectively reduced by grinding aids [14].

A special case of applying additives during the comminution process is the so-called co-milling. Thereby, excipients such as flow additives and lubricants are added to the grinding process in order to coat the product particles simultaneous to the size reduction. The major aim of this process is to adjust the bulk and flow behavior of the ground product [31,32] or to enhance the solubility of the host particles [33,34] rather than to optimize the grinding process. How far the additive addition influences the size reduction is therefore almost unknown. Wang et al. [35] reported that excessive additive amounts may also have negative impacts on the size reduction. Stand and Steckel [36] also revealed positive effects regarding the product particle sizes. Generally, the coatings do not only improve the powder properties, but also passivate the high-energy surface sites of the host particles [32,36]. Thus, positive impacts of these additives on the size reduction, at least when being applied in appropriate amounts, are also very likely.

However, when applying additives to improve specific material properties and process performance in a single unit operation, their effect on downstream unit operations must be taken into account. In general, the intended effects of additives on (intermediate) product properties conflict for milling and subsequent compaction processes. The former needs to separate the particles to avoid agglomeration and the latter has the predominant aim to agglomerate particles to yield one solid product with sufficient strength for further handling. Nevertheless, a few recently published studies reveal that dry coatings do not affect the powder properties as well as subsequent process units such as tableting. According to Kunnath et al. [37], the tablet strength can be improved by flow additives. Thereby, hydrophilic guest particles as well as guest particles with smaller size are favorable. In contrast, Chattoraj et al. [38] found a decreased tensile strength for dry coatings with nano-silica. Coatings made of lubricants such as magnesium stearate lead to reduced tensile strength of the tablets [39,40]. 

The present studies consider in detail the effect of grinding aids on product properties in subsequent process steps. For the first time, this is shown in a systematic manner using the example of a whole process chain comprising fine comminution and subsequent tableting. The results reveal a significant need for further investigation. On the one hand, how liquid grinding aids affect the fine grinding of organic materials in terms of processability, process efficiency and product qualities, is almost unknown. On the other hand, the functional mechanisms of most additives—for both grinding aids as well as common solid additives—are not fully understood, especially in terms of opposing effects during the different process operations such as milling and compaction. For this reason, we investigated the impact of various additives on the fine grinding of different organic model materials within a fluidized bed-opposed jet mill and evaluated their impacts on the resulting particle and powder properties as well as on the subsequent tableting of the ground material.

## 2. Materials and Methods

### 2.1. Material

In this study, two pharmaceutical excipients, α-lactose-monohydrate (CapsuLac60, Meggle, Wasserburg am Inn, Germany) and microcrystalline cellulose (Parmcel 102, Gustav Parmentier, Frankfurt am Main, Germany), as well as the model active pharmaceutical ingredient (API) theophylline, anhydrous (Acros Organics, Geel, Belgium), were used for the grinding and tableting experiments (see Table 1).

Furthermore, two liquid and two particulate chemicals from different substance classes were used as additives (see Table 2). While the liquid additives are established as grinding aids for processing inorganic materials, the particulate additives are typically applied as flow aids (nanoscale silicon dioxide) or lubricants (sodium stearyl fumarate). In these experiments, the concentrations were chosen between 1 and 5 wt.% for the particulate and at 0.1 wt.% for the liquid additives respectively, related to the total powder mass. These concentrations correspond to typical additive amounts for processing particles in the lower micron scale.

### 2.2. Methods

#### 2.2.1. Processing

##### Blending

To ensure a homogenous dispersion before the milling process, the formulations of raw material and additive were mixed in a three-dimensional blender (TURBULA T2F, WAB-Group, Muttenz, Switzerland). Therefore, the mixture was processed for 20 min with a speed of 72 rpm.

##### Micronization

A fluidized bed-opposed jet mill with an integrated classifier (Picojet, Hosokawa Alpine, Augsburg, Germany) was used for the grinding experiments. To investigate the impact of the additives on the comminution, both the pure materials (without additive) and the pre-blended formulations were micronized at a constant air pressure of 4 bar. In order to reach a median particle size of x_50,dry_ ≈ 5 µm, different classifier speeds were chosen for each material (lactose 18,000 rpm, MCC 45,000 rpm, theophylline 20,000 rpm). The classifier speed for lactose and MCC was kept constant for the pure materials and their formulations. To achieve a comparable particle size of x_50,dry_ ≈ 5 µm, the classifier speed had to be reduced for the theophylline formulations to values of 15,000–18,000 rpm. During the micronization, the feed rate was adjusted manually to achieve a constant power draw of the classifier, which was determined as 1.2-fold that of the no-load power. For each sample, 100 g were micronized, and the grinding time was quantified to calculate the product throughput. After micronization, the amount of product particle adhesion in the classifier area was removed and weighed.

##### Tableting

The raw (blended) and ground samples were compacted with the compaction simulator Styl’One Evolution (Medel’Pharm S.A.S., Beynost, France) using Euro-D punches and dye (Adamus, Szczecin, Poland) to produce 10 cylindrical tablets with a diameter of 11.28 mm for each sample and process conditions. A symmetrical movement of the punches with a speed of 35 mm/s in the linear range and a dwell time (punch holding time) of 20 ms were adjusted for the compaction. Due to the poor processability of the samples, the dye-wall was lubricated with magnesium-stearate and 300 ± 5 mg of the respective powder was manually filled for each compaction. In this study, three different compaction pressures, 100, 200 and 400 MPa, were investigated for each sample. Furthermore, the recorded data were analyzed with the software Analis version 2.08.3 (Medel’Pharm S.A.S., Beynost, France) to receive information regarding the energy conditions and the in-dye porosity. Then, all the final tablets were tested with regard to their mechanical properties (see Mechanical Properties of Tablets).

#### 2.2.2. Analysis

##### Particle Size Distribution

The particle size distributions of the ground samples were determined through dry measurements by the laser diffraction particle size analyzer Helos. The dosage unit Vibri, which consisted of a funnel and a vibratory channel, was used to transport the particles to the dry dispersion unit RODOS (all devices, Sympatec, Clausthal-Zellerfeld, Germany) continuously during the measurement. Within RODOS, the particles were dispersed under an air pressure of 2.0 bar to achieve a compromise between sufficient dispersion and avoidance of particle fragmentation.

##### Specific Surface Energy

The specific surface energy of the raw samples (without additives) and the ground samples was determined by inverse gas chromatography (iGC) with a surface energy analyzer (iGC-SEA) (Surface Measurement Systems, London, UK). For this measurement technique, a dry powder sample is constantly flowed through by an inert carrier gas flow (helium). In order to determine the sample surface properties, a known volume of different gas molecules is injected to the carrier gas flow. These gas molecules interact differently with the sample surface via adsorption. More information regarding the measurement principle can be found in [41]. In this study, the powder sample was filled into a silanized glass column with a diameter of 2 mm. In order to achieve a homogeneous well-consolidated packing, the filled column was tapped for 5 min in a tap device. Prior to analyzing, the sample column was conditioned for 6 h at a temperature of 50 °C. Then, the dead volume of the packed column was quantified by adding methane injections. In order to measure the BET-surface of the powder sample, multiple octane injections with a relative gas load between 0.05 and 0.35 were added to the carrier gas. Then, the surface energy was determined by different non-polar (n-decane, n-nonane, n-octane) and polar (toluene and chloroform) molecule injections within a surface coverage range between 0.5% and 14% at a column temperature of 30 °C. For the evaluation, the elution peaks were quantified by their center of mass to determine the retention time, $t_{R}$, and the helium gas net retention volume, $V_{R}^{0},$ (see Equation (1)):

(1) $$V_{R}^{0}=\frac{j}{m}\cdot F\cdot \left(t_{R}-t_{0}\right)\cdot \frac{T}{273.15}$$

where j is the James–Martin correction, m is the sample mass, F is the helium volume flow rate, $t_{0}$ is the dead time of the helium flow and T is the column temperature. Additionally, the helium gas net retention, $V_{R}^{0}$, is defined as (see Equation (2)):

(2) $$-\Delta G^{0}=R\cdot T\cdot lnV_{R}^{0}+k$$

with the Gibbs free energy of adsorption, $-\Delta G^{0},$ the universal gas constant, R, and a constant related to the reference state, k. Furthermore, the approach of Schulz was used in order to calculate the surface energy, γ. Due to the energetic heterogeneity of the particle surfaces, $\gamma_{90}$ was chosen for the characterization of the surface energy, where $\gamma_{90}$ represents 10% of the particle surface, which has an equal or a higher surface energy than the $\gamma_{90}$ value. In accordance with Fowkes [42], the attractive forces on the particle surface and hence the particle surface energy can be divided into two parts, the London’s dispersive interactions and the specific acid–base interactions (see Equation (3)):

(3) $$\gamma^{T}=\gamma^{D}+\gamma^{AB}$$

where $\gamma^{T}$ is the total surface energy, $\gamma^{D}$ is the dispersive part and $\gamma^{AB}$ is the specific part of the surface energy. Due to the high time required for the test procedure, each sample was only measured once.

##### True Density

The true density of the raw materials was measured by the helium pycnometer Ultrapyk 1200 e (Quantachrome Instruments). For each sample, the average of ten consecutive volume measurements was calculated.

##### Powder Flowability

The powder flowability of the samples was measured before and after micronization by a ring shear tester RST-XS (Dietmar Schulze Schüttguttechnik, Germany). The measurements were performed in a 30 mL shear cell under ambient conditions. Each sample was analyzed two times at minimum. In the beginning of the shear test, the powder sample was pre-sheared under a normal stress, $\sigma_{pre}=5$ kPa, until a constant shear stress, $\tau_{pre}$, was reached. Then, the sample was gradually sheared under lower normal stresses, $\sigma_{she}$ ($\sigma_{she,1}=1.25$ kPa, $\sigma_{she,2}=2.5$ kPa, $\sigma_{she,3}=3.75$ kPa), as long as incipient flow was achieved ($\tau_{she})$. In order to obtain a comparable consolidation state, the sample was pre-sheared with $\sigma_{pre}=5$ kPa after each normal stress, $\sigma_{she}$. Each pair of normal stress and shear stress (e.g., $\sigma_{she,1},\;\;\tau_{she,1})$ was used to construct the yield locus. Based on the Mohr stress circles, the major principal stress, $\sigma_{1}$, and the unconfined yield strength, $\sigma_{c}$, were determined. In this study, the flowability index, $ff_{c}$ (see Equation (4)), was used to characterize the powder flowability:

(4) $$ff_{c}=\frac{\sigma_{1}}{\sigma_{c}}$$

In accordance with Jenike, the $ff_{c}$ can be used to classify the powder samples in different groups related to their powder flowability (see Table 3).

##### Bulk and Tap Density

The bulk (ρ_b_) and tap density (ρ_t_) of the samples were determined before and after the micronization according to the EUP (European Pharmacopoeia [44]). To ensure reproducible results, the samples were loosened by sieving the powder (mesh size 1 mm) prior to the measurements to break up agglomerates, which might have formed during storage. For the bulk density, a sample volume, V_0_, of 90 mL was filled in a graduated cylinder in order to measure the related mass. The bulk density was calculated from the ratio of mass and volume, V_0_. To obtain the tap density of the samples, the graduated cylinder containing the untapped sample was tapped mechanically in a tap density tester (Erich Tschacher, Laboratoriumsbedarf Bielefeld, Bielefeld, Germany). The sample volumes V_10_, V_500_ and V_1250_ were determined after 10, 500 and 1250 taps. If the difference between V_500_ and V_1250_ was more than 1 mL, the sample was stressed with an additional 1250 taps until the volume difference was 1 mL or less. The tap density was calculated from the ratio of the mass and the final tap volume, V_t_. Each sample was analyzed three times to receive a statistical reliability.

##### Particle Morphology

The morphology, size and the agglomeration status of the raw samples (without additives) and the ground samples were analyzed by a scanning electron microscope (Helios G4 CX Dual Beam, Thermo Fisher, Waltham, USA). In order to avoid electrical charge, the samples were sputtered with a thin layer of 6 nm platinum before analyzing.

##### Mechanical Properties of Tablets

To characterize the final tablets, geometric properties, such as diameter, $d_{tablet}$, and thickness, $h_{tablet}$, the breakage force, F, and the weight, m, were determined 24 h after compaction. The geometric properties and the breakage force were measured by the tablet hardness tester MultiTest 50 (Dr. Schleuniger Pharmatron, SOTAX AG, Basel, Switzerland). For each sample, 10 tablets were tested, and the average was calculated. The tensile strength, $\sigma_{o}$, for cylindrical tablets was determined by Equation (5) [45]:

(5) $$\sigma_{0}=\frac{2F}{\pi \cdot d_{tablet}\cdot h_{tablet}}$$

Furthermore, the tablet porosity, ε, for a cylindric tablet was quantified by Equation (6), whereby $\rho_{tablet}$ is the final tablet density and $\rho_{material}$ is the material density:

(6) $$\varepsilon =1-\frac{\rho_{tablet}}{\rho_{material}}$$

The final tablet density, $\rho_{tablet}$, is given as (see Equation (7)):

(7) $$\rho_{tablet}\;=\;\frac{m_{tablet}}{\frac{\pi }{4}\cdot d_{tablet}\cdot h_{tablet}}$$

## 3. Results and Discussion

### 3.1. Fine Grinding

#### 3.1.1. Micronization without Additives

**Figure 1:**
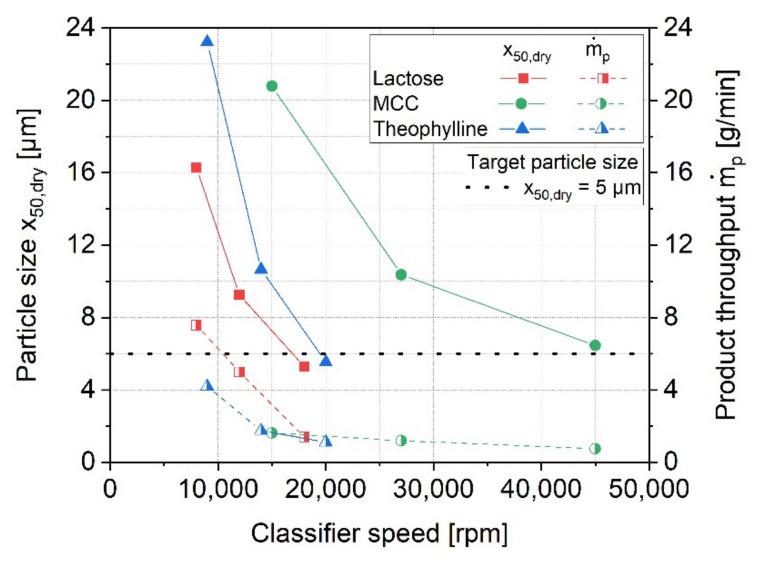
Particle size, x_50,dry_, and product throughput, $\dot{m_{p}}$, as functions of the classifier speed using a fluidized bed-opposed jet mill at an air pressure of 4 bar.

In a first instance, the micronization behavior of the different materials was investigated in the fluidized bed-opposed jet mill without the addition of any additive. Figure 1 shows the resulting median particle sizes as well as the product throughputs for a constant air pressure of 4 bar and different classifier speeds. According to the figure, an increase of the classifier speed leads to a reduction of the particle size, x_50,dry_, for all materials. At the same time, clear differences are seen regarding the specific classifier speeds, which are required to obtain certain particle sizes. Theophylline and MCC require slightly and significantly higher classifier speeds respectively, to achieve a similar product fineness in comparison to lactose. A reduction of the product particle size leads to decreasing product throughputs, independent of the material. Therefore, much higher production capacities are reached for the grinding of lactose, at least in the range of relatively coarse product particles.

A decrease of the production capacity is generally not surprising when aiming for finer products, since higher specific energies are needed to generate the new surfaces. In addition to that, the number of defects inside the particles is normally reduced with decreasing particle size, which makes finer particles even more difficult to break [46,47,48]. In the present study, the air pressure, and thus, the energy input into the mill, was kept constant during the micronization. Higher fineness values are therefore primarily achieved by multiple stressing of the particles, requiring longer retention times of the material inside the mill. As the material loading inside the mill is in turn limited by the power draw of the classifier, this automatically affects lower product capacities. 

**Figure 2:**
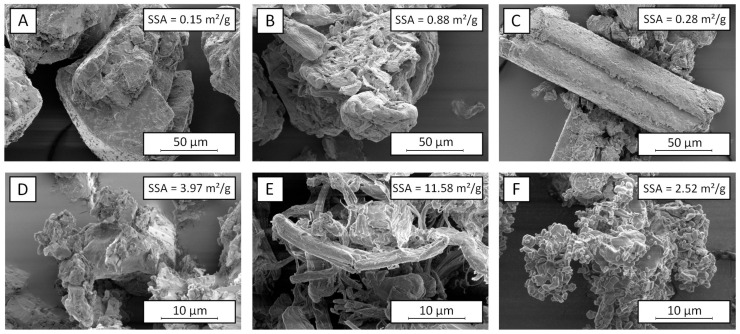
SEM images of raw materials and materials ground to an x_50,dry_ of approximately 5 µm and their specific surface areas (SSA) calculated from octane-BET measurement using iGC: (**A**) Raw lactose, (**B**) raw MCC, (**C**) raw theophylline, (**D**) ground lactose, (**E**) ground MCC, (**F**) ground theophylline, un-stabilized, respectively. Magnifications of 2000× (**A**–**C**) and 10,000× (**D**–**F**), respectively.

Generally, a decrease of the particle size through increasing the classifier speed is prevalent for the classification in a rotational flow field. The particles are affected by a centrifugal force within the rotational flow field, which counteracts the drag force of the air flow. Since higher centrifugal forces are therefore acting on large particles, coarse particles above a certain cut size are primarily deflected at the classifier wheel, while fine particles are transported through the classifier wheel by following the air stream. During the tests, the air stream, and thus, the drag forces of the air flow, remained constant, which is why an increase of the classifier speed leads to higher centrifugal forces and a reduction of the product particle size. Due to the similar solid densities of the materials (Table 1), comparable product particle sizes would have been expected for similar classifier speeds (see, e.g., [49]). It is therefore assumed that differences of the particle structures and morphologies led to the observed changes of the particle behavior within the classification zone. As shown in Figure 2, there are apparent differences of the particle morphology for the investigated materials. Already, the raw materials show different shapes and structures, whereby rather compact particles with angular shapes can be seen for lactose and theophylline. In contrast to that, the MCC particles have a less defined, filamentary structure, with higher porosity. These differences are also found for the micronized samples. While fine grinding of lactose and theophylline leads to small but compact fragments, which tend to form agglomerates due to high particle–particle interactions, the filamentary and porous structure of MCC is further intensified by stressing the particles. Most likely, the morphology of the MCC particles is responsible for the different classification behavior in the rotational flow field of the classifier wheel. Due to the high porosity of the particles, the actual particle density is much lower than the density of the solid material. In comparison to lactose and theophylline, higher centrifugal forces, achieved by using higher classifier speeds, are necessary to deflect particles of an identical apparent size. Additionally, the porous structure of the ground MCC leads to high specific surfaces of 11.58 m^2^/g, which is 3- to 4-times higher compared to the specific surface areas of the ground qualities of lactose and theophylline.

The particle morphologies of the ground lactose and theophylline show further differences. While lactose builds edged particles with an irregular surface, the fragments of theophylline have a rather round and smooth shape. Furthermore, the SEM image of the ground lactose indicates the presence of finer fragments, which would explain its higher specific surface area. This in turn was not seen during the particle size analysis, probably due to a strong adhesion of the small fragments onto the surface of larger particles. Generally, the different breakage behaviors are mainly related to a more brittle material behavior of lactose in comparison to theophylline [46]. These results are confirmed by the compactions results, as Table 4 clearly shows less elastic deformation for lactose, whereas theophylline is deforming more elastically. It is assumed that the brittle fracture enables a higher utilization of the applied stress energy for breakage instead of plastic deformation, leading to both a higher process efficiency (see the higher product throughputs of lactose) and to the generation of finer fragments. In contrast, more ductile particles undergo plastic deformation during stressing, which may counteract an efficient breakage due to higher energy consumptions. In contrast to lactose and theophylline, the impact stressing of MCC particles results in a dispersing of the filamentary structures, rather than in a real particle breakage. It can be assumed that the generated cellulose filaments undergo a strong plastic deformation during stressing due to the deformation behavior of cellulose. Thus, generally, lower parts of the applied stress energy are available for real particle breakage, which further accounts for the generally low product throughputs of MCC.

**Figure 3:**
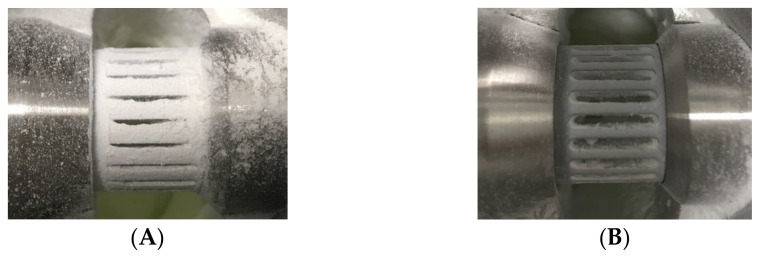
Product particle adhesion of theophylline at the classifier wheel after micronization: (**A**) without additives and (**B**) with 1 wt.% SSF.

In the case of theophylline, the increased plastic deformation of the compact particles may also lead to larger contact areas between the particles, resulting in higher adhesion forces, and thus, the formation of even stronger agglomerates. This is supported by larger amounts of comparatively strong theophylline adhesion, which were found on the mill equipment after the grinding. On the one hand, the formation of strong agglomerates counteracts a high energy utilization during the particle stressing, as the compaction and breakage of agglomerated particle structures consumes parts of the kinetic grinding energy, which is then not available for the breakage of primary particles anymore. On the other hand, the formation of agglomerates also affects the particle classification inside the mill. In the case of a strong agglomeration tendency, fine particles are increasingly deflected at the classifier wheel as agglomerates, and led back to the grinding zone. This does not only decrease the product throughput, but also reduces the number of agglomerates inside the mill, which in turn makes the particle stressing in the grinding zone less efficient. Finally, this leads to a reduced product throughput as well as lower grinding efficiencies of the mill. The aforementioned theophylline adhesions were detected especially at the deflector wheel of the classifier, leading to a partial blocking of the gaps inside the wheel during the processing without any additives (Figure 3A). Consequently, highly unstable processing conditions were seen as the clogging of the gap changed with the grinding time. Additionally, the product quality was reduced (e.g., due to broad size distribution) due to changes of the classifying behavior. It needs to be noted that such a behavior is normally critical for industrial processes. It would therefore limit the micronization of such materials in jet mills.

#### 3.1.2. Micronization with Additives

**Figure 4:**
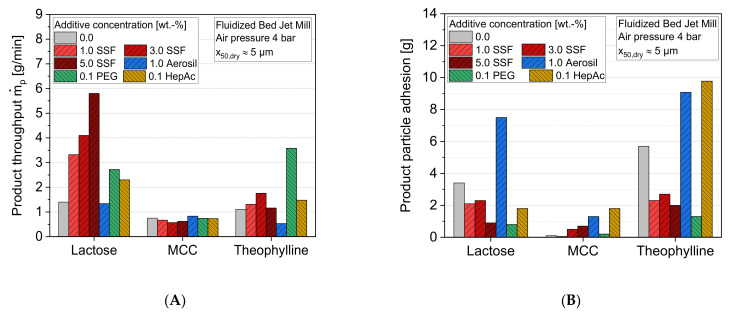
Impact of additive and additive concentration on (**A**) the product throughput, $\dot{m_{p}}$, and (**B**) product particle adhesions, *n* = 1.

In order to characterize the influence of the additives on the micronization process inside the jet mill, both the product throughput as well as the loose particle adhesions in the classifier zone were investigated for a nearly constant product particle size of x_50_ ≈ 5 µm. Figure 4A shows the impact of the additives on the product throughputs in comparison to the grinding without additives, revealing several material-specific additive effects.

While most of the additives, both particulate and liquid, show a positive influence on the product throughputs for lactose and theophylline, there are no significant changes for MCC. For the former two, SSF and PEG lead to the highest product throughputs, while the addition of HepAc results in smaller improvements. In contrast, the solid flow additive Aerosil has no (lactose) or even a negative (theophylline) influence on the production capacity. Furthermore, the results of lactose imply a strong dependence on the additive concentration. Here, the product throughputs increase significantly for a higher SSF concentration. In contrast, an optimum SSF concentration of 3 wt.% is found for theophylline. The different optimum concentrations are not surprising, since theophylline shows generally lower specific surface areas after the grinding. It can be assumed that the adsorption (e.g., liquid grinding aids) or adhesion (e.g., solid additives) of the additives on the particle surface leads to a reduction of the particle–particle interactions, and thus to a reduced particle agglomeration. Thus, generally lower additive amounts are needed in the case of smaller specific surfaces.

**Figure 5:**
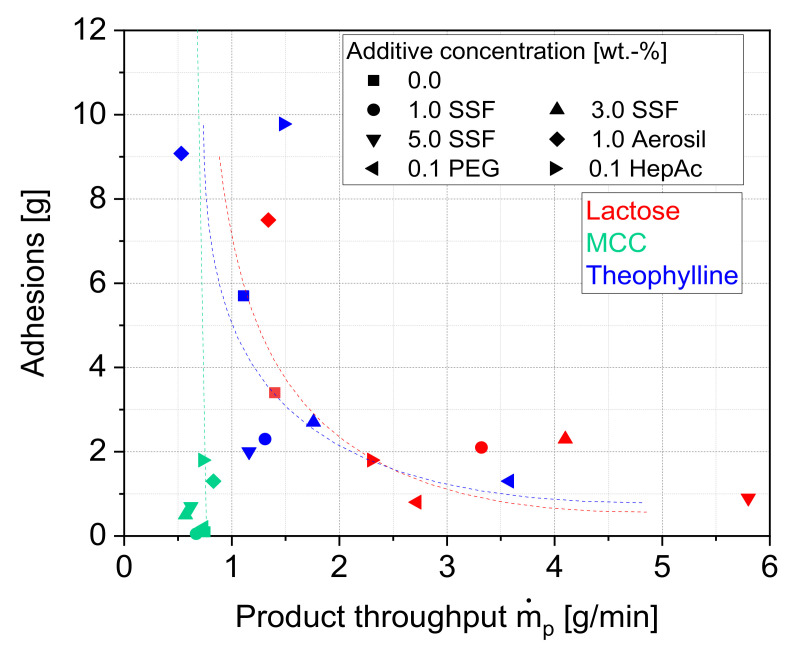
Relation between product throughput and product particle adhesion.

This assumption is supported by the amounts of product adhesions inside the mill (Figure 4B). These adhesions can be used as a surrogate for describing particle–particle interactions. A comparison of Figure 4A,B reveals a roughly inverse correlation between the product throughputs and the loose particle adhesions, as Figure 5 depicts: an efficient minimization of product particle adhesions inside the mill leads to highest throughputs. With regard to this type of mill, the adhesions will change the flow regimes inside the mill, which influences the grinding and therefore the efficiency of the process in a negative way. It clearly shows that the grinding process as well as the formation of product coatings is influenced by a similar stabilization mechanism. Thereby, additives such as lubricants (e.g., SSF) or grinding aids (e.g., PEG, which is also generally usable as a lubricant) are very effective in terms of controlling the agglomeration behavior inside the mill, leading to both a reduction of adhesions inside the mill and a higher energy utilization during the micronization. Depending on the material, the particle stabilization improves the grinding process in terms of the aforementioned aspects. However, the varying additive effects underline, again, that the extent of the particle stabilization is not solely determined by the additive class itself, but rather by the combination of a ground material and stabilizer.

**Figure 6:**
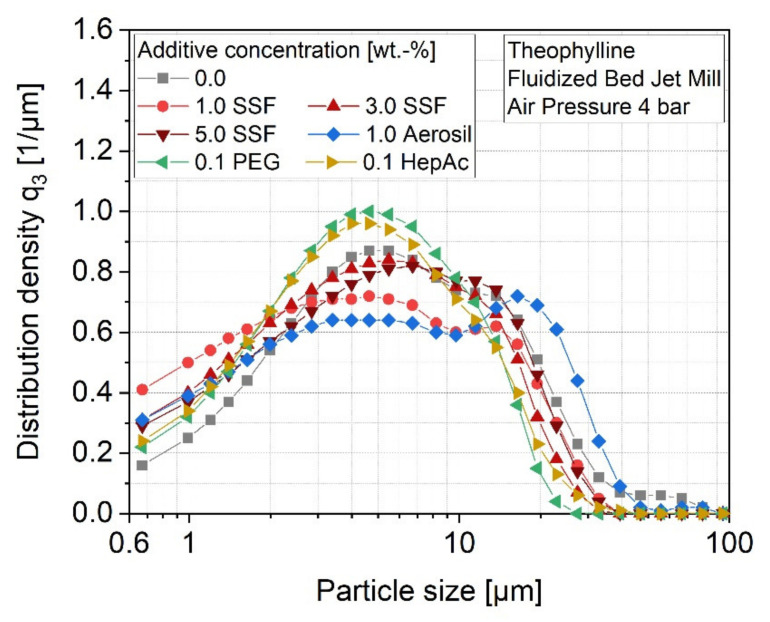
Impact of additives and additive concentration on the distribution density of theophylline. In order to achieve a median particle size x_50,dry_ = 5 µm, the classifier speed, n, was adapted: n_0.0_ = 20,000 rpm, n_SSF_ = 15,000 rpm, n_Aerosil_ = 18,000 rpm, n_PEG_ = 18,000 rpm, n_HepAc_ = 16,000 rpm.

In extreme cases such as theophylline, the prevention of agglomeration is essential for maintaining stable processing conditions for a further reason; as described before, significantly larger material adhesions are found in the case of theophylline due to comparatively high inter-particulate forces of this material. In this regard, also the strength of the adhesions on the surfaces needs to be considered. While most of the bound material could be easily removed from the surfaces by brushing, grinding of theophylline without any additive or with Aerosil also resulted in strongly adhered material layers directly at the classifier wheel (compare Figure 3). On the one hand, strong agglomerates consume even more energy inside the grinding zone, leading to a further decrease of the energy utilization during grinding. On the other hand, a clogging of the classifier channels causes an inhomogeneous air flow through the wheel, which results in a poor classifying behavior, and thus, a lower product quality. It is therefore not surprising that the theophylline samples contain drastically larger fractions of coarse particles after grinding without an additive or with Aerosil (Figure 6). Moreover, the clogging of the channels happens over time, which makes the process highly unstable. It further complicates the process control, as the classifier speed needs to be adapted regularly in order to maintain a constant product quality. For materials such as theophylline, which tend to form high adhesion forces, the prevention of these strong material adhesions is of major importance.

Interestingly, the formation of these strong adhesions was not only prevented by additives such as SSF and PEG, but also by HepAc. This also explains why HepAc slightly improved the product throughput of theophylline, even though it increased the amount of loosely bound adhesions (Figure 5). This finding emphasizes that the strong adhesions generally illustrate a more critical agglomeration behavior than the amount of loosely bound particles described before.

**Figure 7:**
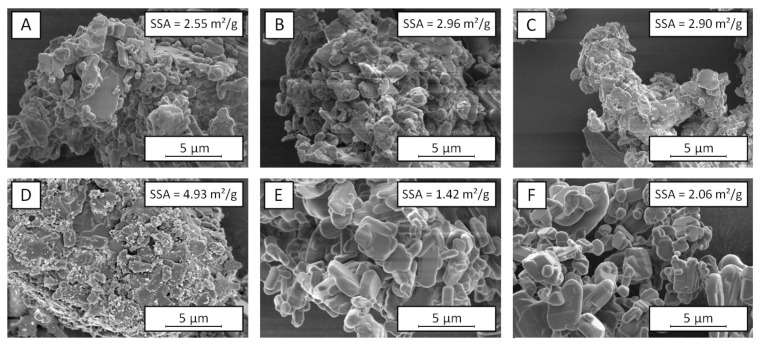
SEM images of ground theophylline (to a final particle size of x_50_ = 5 µm) with different additives and their specific surface areas (SSA) calculated from octane-BET measurements using iGC: (**A**) stabilized with 1.0 wt.% SSF, (**B**) stabilized with 3.0 wt.% SSF, (**C**) stabilized with 5.0 wt.% SSF, (**D**) stabilized with 1.0 wt.% Aerosil, (**E**) stabilized with 0.1 wt.% PEG, (**F**) stabilized with 0.1 wt.% HepAc. Magnification 20,000×, respectively.

Throughout this study, the application of nanoscale flow additives such as Aerosil did not lead to comparable benefits. Neither the efficiency of the grinding process was increased, nor were the product adhesions reduced. In fact, even higher amounts of loose adhesions were observed, which is why it cannot be ruled out that the silica particles even act as a bonding agent towards the metallic mill surfaces. The missing effects regarding the grinding efficiency can be explained by considering the SEM images of the ground samples, which are shown exemplarily for theophylline in Figure 7. In the case of Aerosil, the additive particles are clearly visible on the particle surface, which obviously reveals an insufficient surface coverage. Thus, an insufficient surface coverage may also be responsible for the missing stabilization effect.

In the other cases, it is difficult to assess whether the additive molecules are distributed over the particle surface more homogeneously. From micronization of inorganic materials, it is known that liquid additive molecules adsorb preferentially via their polar, functional -OH groups on the particle surface, leading to monomolecular films already at comparatively low additive concentrations. Thereby, both the number of functional groups and the arrangement in the additive molecule determine the number of molecules, which is needed to saturate the particle surface [14]. This relation can be seen in the different efficiencies of the stabilization effect by PEG and HepAc. Due to the high number of functional groups within the PEG molecule, it is most likely that PEG covers the particle surface over a larger area, whereas HepAc adsorbs selectively to the surface with only one functional group. From the field of flow additives, it is further approved that the nanoparticulate additive Aerosil adsorbs via van der Waals forces on the particle surface [31]. This mechanism is in accordance with the SEM image depicting Aerosil particles on the surface (see Figure 7D). In case of SSF, it is most likely that thin layers of SSF particles are sheared off during the micronization process, which adsorb on a large area and, in this way, a great fraction of the particle surface. However, this assumption is rarely reasonably provable solely by SEM images, as also, if coupled with EDX, SSF is under the detection limit (data not shown). This is due to the only marginal differences in atomic composition of the contributing molecules.

These assumptions imply that especially those additives leading to a high degree of surface coverage (e.g., SSF and PEG) achieve an improvement of the micronization process and, thus, an effective stabilization. Therefore, even a thin but rather complete adsorption layer appears to be of high importance in order to avoid a direct contact of the particles while stressing. The thickness of the additive layer (e.g., as seen for the large Aerosil agglomerates) appears to be less important, as long as large surface areas are not covered by the additive.

Furthermore, the different impacts of SSF within the investigated dosage range show that the additive concentration is also important for an effective particle stabilization. A low additive concentration results in an incomplete coverage of the particle surface, which is why the full potential of the additive is not reached yet. In contrast, an “over-concentration” of the additive can also lead to a reduction of the additive effect. In this case, a thick and complete adsorption layer of multiple layers of particulate additives could function as an impact damping. As a consequence, the energy transferred by stress is not fully used for particle breakage, but for elastic–plastic deformation of the adsorption layer. In general, it is difficult to find an optimal concentration for effective stabilization ab initio. The optimal concentration is primarily determined by the specific surface area of the material and the additive-specific adsorption/adhesion mechanism on the solid surface.

The missing effect of the additives for MCC can be explained by two different effects. First, no real particle breakage, but a dispersion of the filamentous structure, occurs during the micronization. Thus, any stabilizing effects by applied additives are rather low and overlapped by the more decisive material behavior. Secondly, a too-low surface coverage, which may originate from the high specific surface areas of MCC, that are further drastically enlarged by milling, can also be the reason for the missing effects. However, since the increase of the SSF concentration did not lead to any improvements either, the former explanation seems more decisive. This indicates that such additives generally show low benefits in terms of improving the processing in jet mills for certain classes of ground products.

### 3.2. Effects on Particle and Bulk Properties Relevant for Further Processing

#### 3.2.1. Specific Surface Energy

**Figure 8:**
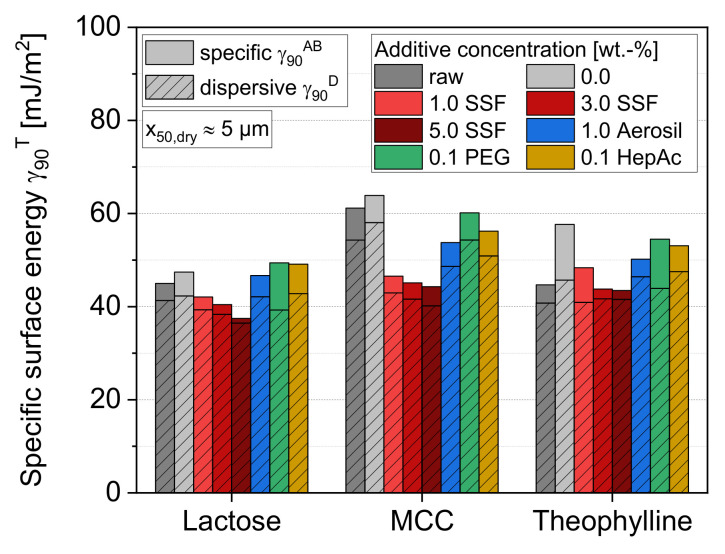
Impact of additive and additive concentration on the specific surface energy of raw particles and milled particles (x_50,dry_ approximately 5 µm), *n* = 1.

Already, the raw materials show differences regarding their specific surface energies (Figure 8). While lactose and theophylline have similar $\gamma_{90}^{T}$ values, the total specific surface energy for raw MCC is significantly higher due the highly dispersive part, $\gamma_{90}^{D}$. This finding is in accordance with Swaminathan et al., who showed that, despite the largely crystalline character of MCC, the highly dispersive component is caused by the small amorphous part of MCC [50]. In general, all materials show a drastically higher dispersive ($\gamma_{90}^{D}$) than polar ($\gamma_{90}^{AB}$) surface energy. This behavior is different to studies of inorganic materials, where much higher ratios of the specific to dispersive energy component were determined [14].

Furthermore, the results show that the micronization without any additives leads to a slight (lactose and MCC) or even stronger (theophylline) increase of the total specific surface energy. In all cases, the dispersive surface energy slightly increases during grinding. For theophylline, the stronger growth of the total surface energy, $\gamma_{90}^{T}$, is mainly based on an additional increase of the specific polar part, $\gamma_{90}^{AB}$. Depending on the product, the higher surface energy values are known to be caused by generating amorphous surface regions [51] or by uncovering new fracture surfaces with different surface energies [52]. This supports the finding that theophylline led to high and resilient product adhesion in the milling process without an additive. By applying the liquid grinding aids PEG and HepAc, or the solid flow aid Aerosil, no pronounced (lactose) or only slight (MCC and theophylline) reductions of the total surface energy are achieved, in comparison to grinding without additives. In contrast, a moderate to strong reduction is obtained by the additive SSF. The effects slightly increase for higher SSF concentrations, underlining the assumptions of higher surface coverage. Thereby, the surface energy values approach similar total values in the range of 40 mJ/m^2^ for all materials, including very low and concentration-dependently decreasing polar surface energies. It is assumed that the measured surface energies become closer to the values of the pure SSF, as the ground particles are increasingly covered with additive layers. Thus, the maximum effect in terms of energy reduction is limited by the energetic properties of the additive itself. With the liquid additive PEG, where a nearly complete surface coverage is also likely, at least for lactose and theophylline, slightly higher total values of 47–50 mJ/m^2^ can be seen. In contrast, Aerosil and HepAc show generally high values due to higher-energetic surface sites, which are still located at the surface at incomplete particle coverage.

#### 3.2.2. Powder Flowability

**Figure 9:**
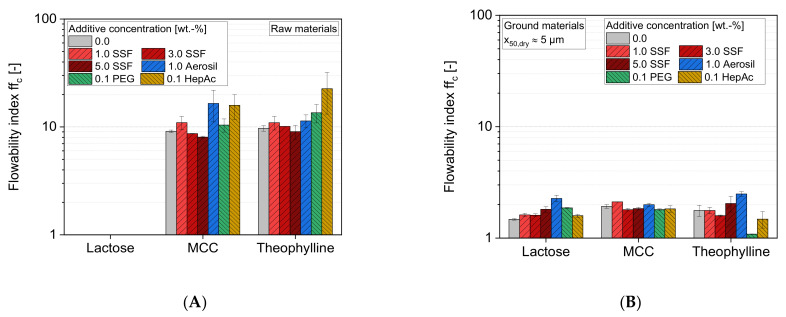
Impact of additive and additive concentration on the flowability index (*ff_c_*) for (**A**) raw and (**B**) ground materials. Due to the high flowability of raw lactose (*ff_c_* >> 10), no reliable values were obtained, *n* = 3.

Due to the high flowability of raw lactose ($ff_{c}>>10$), no reliable $ff_{c}$ values could be obtained for these formulations. According to Figure 9A, raw MCC and raw theophylline can be classified as easy-flowing powders ($4<ff_{c}<10),$ with $ff_{c}$ values of approximately 9. The powder flowabilities are slightly increased, especially by HepAc, changing the flow behavior of the sample from easy-flowing to free-flowing $\left(ff_{c}>10\right)$. Aerosil shows a material-specific behavior, as a drastic effect is only seen for MCC. Interestingly, SSF and PEG do not show strong effects, even though high surface coverages can be assumed for these additives. The results of SSF further emphasize that excessively high concentrations can lead to a reduced flow behavior. In both cases, the addition of 1% SSF slightly increases the powder flowability. In contrast, the higher concentrations (3% and 5%) tend to slightly decrease the $ff_{c}$ value again. 

A comparison of the flowabilities of raw and ground materials shows that the micronization leads to a decrease of the flowabilities (Figure 9B). With $ff_{c}$ values of around 1.5 to 2.0 for the samples without additives, the flow characterization can be determined as very cohesive (MCC) and even not flowing (lactose, theophylline). In contrast to the influences of the additives on the raw materials, most of the additives do not significantly change the flow behavior of the ground samples. Only the additive Aerosil leads to a slight increase of the $ff_{c}$ value > 2 for lactose and theophylline, and therefore, to a rather cohesive powder flow behavior for these samples. The strong decrease of the $ff_{c}\;$ values for theophylline samples containing the liquid additives PEG and HepAc is also very remarkable. In this case, an “over-dosing” of the additive is not very likely due to the high product surface. Most likely, the low flowability is rather a consequence of other parameters, such as the particle size distributions of the ground product, which differ, especially for PEG and HepAc (compare Figure 6), or stronger particle–particle interactions due to the comparatively higher surface energies (Figure 8).

#### 3.2.3. Powder Densities

**Figure 10:**
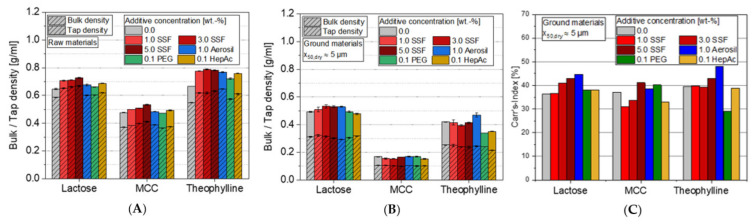
Impact of additive type and additive concentration on the bulk and tap density for (**A**) raw and (**B**) ground materials, as well as (**C**) Carr’s index for ground materials, *n* = 3.

In case of bulk and tap densities of the raw and ground powders (Figure 10), small additive effects can be identified, especially for the raw materials. For each of the three materials, a slight increase of both powder densities can be seen for the lubricant SSF, and also partially for the other additives. The relative differences between bulk and tap density stay mostly in a comparable range. Thus, the corresponding Carr’s indices indicate that the compressibility of the raw powders is not significantly influenced by adding any of the additives (Figure 10C). Independent of the additive, a material-specific change of the Carr’s index can be observed during grinding. Especially for lactose, grinding leads to an increase of the Carr’s index, independent of the additive, and thus to a higher compressibility of the powder. This is in good accordance with the highly decreased flowability of lactose due to grinding. Furthermore, the observed additive effects almost vanish as a consequence of the micronization. Significant additive effects are only seen partially for any of the ground materials. For instance, Aerosil in general or PEG admixed to theophylline show slightly higher and lower Carr’s indices than the sample ground without any additive, respectively. It indicates that the compressibility can be increased (Aerosil) or even reduced (PEG) by adding additives to the grinding process. Interestingly, the index contradicts the flowability measurements from the ring shear tests: since the theophylline sample ground with Aerosil showed a comparably high $ff_{c}$ value (or a very small value for PEG), the Carr’s index provides a controversial impression of the flow behavior. This discrepancy generally emphasizes the compromised informative power of this index, which is not surprising as the samples show further differences in terms of parameters such as particle size distribution and surface energy (see above).

### 3.3. Relation of Surface Energy and Powder Properties

It is well-known for inorganic materials that additives such as liquid grinding aids significantly reduce the surface energies of the product particles, leading to a decrease of the adhesive forces between the particles, and thus, to a change of the powder properties [14]. The stabilizing mechanism is therefore not only achieved by preventing a direct contact of colliding particles, but also by changing the motion behavior of the particles through the mill near or between the grinding equipment [23]. The results of this work clearly show that such additives cause different effects in the case of organic materials. On the one hand, it is clearly shown that the additives reduce the state of particle agglomeration and increase the grinding efficiency. On the other hand, a significant impact on the powder and flow properties is not seen. It can therefore be concluded that—in the case of these organic particles—the additives rather act by reducing the formation of agglomerates as a consequence of preventing direct particle contact, instead of by influencing the flow and bulk behavior inside the mill. The reason for that can primarily be found in the surface energies of such materials. Many inorganic particles show much higher total surface energies, which mainly originate from a significantly higher polar surface character [53]. Since the minimum achievable surface energy is mostly determined by the applied additive, the maximum achievable relative reduction of the surface energy is therefore much higher for most of the inorganic materials. For organic matter, in turn, the relative reduction potential of surface energy is generally much smaller, as the energy values of the additives are in a comparable range to those of the ground materials. As the resulting surface energy influences the inter-particular interactions, the effects of the additives on the powder properties are also less pronounced compared to inorganic materials.

Thus, there is no clear correlation between the surface energy values and the grinding efficiency, as it was shown for grinding of inorganic materials in batch-wise operated media mills [23]. Obviously, the additives are principally capable of increasing the powder flowability, also for organic materials. Especially, the flow additive Aerosil shows such effects, at least when being added in sufficient concentrations. However, the mechanism can be attributed to an increase of the distance between the particles rather than to a reduction of the surface energy. Furthermore, the observed effects regarding the flowability are comparatively small. The question of how much a stronger increase of the flow behavior influences the fine grinding of organic particles—e.g., by adding much higher amounts of flow additives—is still under investigation and should be answered in future publications. Thereby, it should also be investigated whether the flowability is a critical parameter for grinding in jet mills at all. While the relevance of the powder flowability in media mills is a well-known issue (see, e.g., [23,24]), it lacks scientific studies on the most decisive powder properties in terms of micronization with jet mills.

### 3.4. Tableting

#### 3.4.1. Compression Behavior

**Figure 11:**
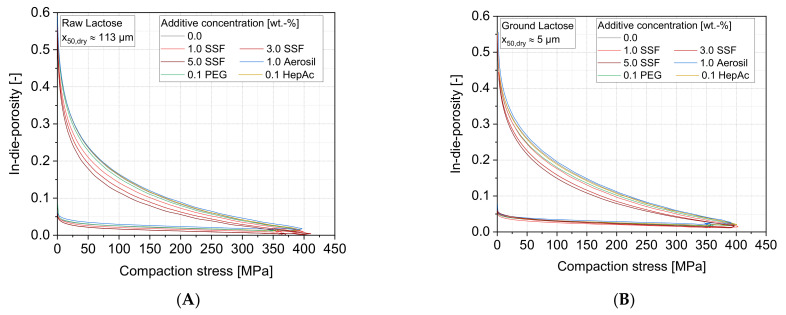
In-dye porosity as a function of the compaction stress for different additives: (**A**) raw lactose and (**B**) ground lactose. *n* = 1 displayed.

The impact of the additives on the compression was analyzed in a compaction simulator. Here, only the results of lactose samples are shown, but similar effects could be observed for all materials. In a first instance, Figure 11 shows typical in-dye porosity curves as a function of the compaction stress for lactose. Thereby, similar additive effects can be seen for both the raw particle size fractions (A) and the ground product (B). As expected from smaller particles sizes that cause lower bulk and tapped densities, the porosities at specific compaction stresses are also higher for ground particles. The results illustrate that the reduction of the in-dye porosity is not or only slightly affected by applying the flow additive Aerosil or liquid grinding aids (PEG, HepAc), respectively. In contrast, the lubricant SSF causes much lower porosities during the compaction, and this effect increases for higher additive concentrations.

The results clearly show that the rearrangement of the particles as a consequence of especially low compaction stresses inside the bulk powder is drastically influenced by the additive SSF. Lubricant particles, which can be assumed to be sheared off along their shear planes under adequate stresses, promote the sliding of the lactose particles, leading to a lower frictional resistance inside the powder. Consequently, the lactose particles can be pushed into the void volume inside the bulk much easier, causing the comparatively steep loss in porosities. The other additives show less pronounced capabilities, even though they partly increased the flow behavior of the loose powder bulk (compare Figure 9, e.g., Aerosil). Most likely, the friction behavior between the particles within loose powder samples at low shear rates is significantly different compared to powders under higher normal stresses at high shear rates. In these cases, a differentiation between shear and rolling motion must be kept in mind. Under such conditions, both incomplete Aerosil coverages as well as thin grinding aid adsorption layers have no or only limited capabilities to maintain low friction forces between the particles, respectively. 

**Figure 12:**
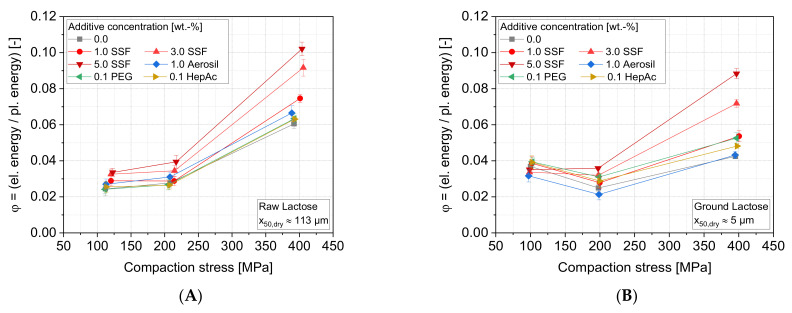
Ratio of elastic to plastic energy, φ, as a function of the compaction stress for different additives: (**A**) raw lactose and (**B**) ground lactose, *n* = 10.

Furthermore, Figure 12 illustrates the impact of the additives on the ratio between elastically and plastically stored energy for three final compaction stresses for lactose. Plastic energy summarizes all fractions of the energy that are dissipated or stored in lasting deformations of the sample. These comprise the mechanisms of particle rearrangement, ductile deformation of materials, such as by contact flattening, as well as brittle fracture of particles. On the other hand, elastic energy comprises elastic deformation mechanisms of the fabric by bending and torsion of particles in the bulk structure as well as the compression of the material itself, i.e., the crystal lattice or unordered, amorphous molecular structure, which both relax during (or after) decompression. For the raw powder samples (Figure 12A), the fraction of the elastically stored energy increases for higher compaction stress. It emphasizes that the formation rate of new contacts between the particles due to plastic (remaining) particle deformation decreases as a denser tablet is obtained, leading to a lower fraction of plastic energy dissipation. Thereby, no drastic difference can be observed between Aerosil, the liquid grinding aids and the sample without any additive. Thus, low effects regarding both the particle rearrangement inside the bulk powder upon compression and the formation of new contact areas are seen for these additives. In contrast, the lubricant SSF shows much higher values of elastically stored energy, especially at high additive concentrations. As the lubricant SSF appears to completely coat host particles (Figure 7) and is able to reduce the surface energy as a function of concentration (Figure 8), it also reduces the adhesion forces between the particles upon deformation and in the tablet. During the unloading of the tablet, higher SSF concentrations will introduce a higher fraction of fragile bonds, causing flaws in the fabric that will break to higher extents upon elastic relaxation. This displays elastic recovery of the compact instead of keeping the energy stored plastically and dissipated as strong inter-particulate bonds. 

In the case of the ground lactose (Figure 12B), comparable additive effects can be observed. A slight decrease of the energy ratio towards lower elastic fractions at lower compression stresses indicates a higher resistance of the bulk against initial deformation, as it is often seen for smaller particles. At 100 MPa, a bulk of small particles provides a higher share of elastic deformation than at 200 MPa. This can be attributed to the higher number of contacts between the smaller and probably stronger particles. When a critical overall stress is exceeded, the stresses in the single-particle contacts are exceeding a critical value to induce lasting deformation by plastic or brittle deformation mechanisms. Accordingly, higher amounts of the introduced energy are dissipated by plastic deformation. At high compression (400 MPa), low porosities, and by that large particle contact areas and low potential for further deformation, lead to an increase of the elastic deformation of the material itself (i.e., crystal deformation or alteration of molecular spacing). Only by addition of higher concentrations of SSF, no minimum at 200 MPa is detected, supporting the hypothesis of the high potential of inter-particulate sliding and damping by high surface coverages of SSF (as presented in Section 3.1.2).

Taking all materials back into focus, Table 4 emphasizes that the compaction behaviors of the three model materials are different, independent of any additives. During the compaction of the micronized powders, much higher energies are stored plastically inside the tablet in the case of MCC, followed by lactose and theophylline. The amount of elastically stored energy is generally much lower than that of plastic deformation for each of the materials. The differences between lactose and MCC can mainly be attributed to the more ductile behavior of the MCC particles. Furthermore, the filamentous and porous MCC particles can undergo a more pronounced plastic deformation until the target compaction stress is reached. The result of the theophylline sample indicates that the material does not only behave as ductile, as already assumed before, but is also comparatively soft. Hence, the target compaction stress is reached without applying large amounts of energy.

#### 3.4.2. Tensile Strength

**Figure 13:**
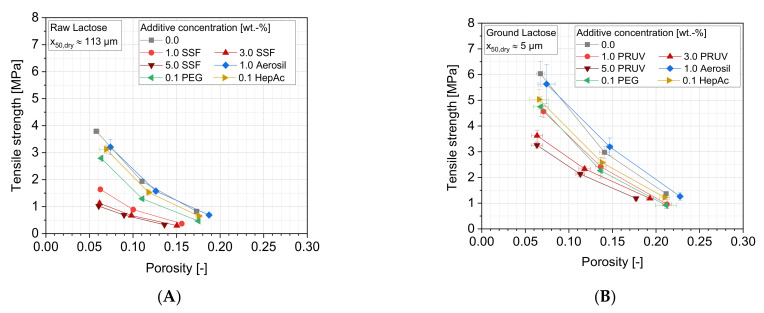
Tensile strength as a function of the tablet porosity for different additives: (**A**) raw lactose and (**B**) ground lactose, *n* = 10.

Figure 13 shows the tensile strengths of lactose tablets as a function of the tablet porosity for the applied additives. The porosity was varied as a result of different defined compaction stresses.

It can be seen that the compaction of the raw lactose (A) yields generally lower tensile strengths compared to the compaction of the ground samples (B), which can be attributed to the (approximately 10,000 times) higher number of particles in the case of a higher powder fineness, leading to a higher number as well as density of bonds inside the tablet. Systematically higher porosities remain for ground lactose, except for the highest compaction stress where comparable porosities are yielded. Additionally, certain additive effects can be observed for both size fractions. While an increasing amount of the lubricant SSF causes much lower tensile strengths of the tablets, the highest strength can be observed for the powders containing Aerosil or no additives. The liquid grinding aids also lead to a reduction of the tensile strength, however, showing much lower effects compared to the lubricant. The decrease of the tensile strength can mainly be attributed to the fact that adhesion forces between the particles are strongly influenced by lubricants and grinding aids. The number of formed bonds will not be highly different (amongst ground or unground samples, respectively), but they can be assumed to be weaker, especially when higher shares of the host particle surfaces are covered with additive molecules or particles. In this way, a direct contact of neighboring lactose particles is partially prevented, and adhesion forces are reduced in a certain fraction of contacts. However, due to its partly brittle deformation behavior (MYP approximately 150 MPa, see Table 4), fresh breakage surfaces, which are not covered by additives, are generated during compaction. These enable a sufficient strength of the tablets and avoid drastic compaction failure, such as by capping or delamination at higher compaction stresses. For SSF, the tensile strength may further be reduced as the surface energies, and thus the particle–particle interactions, are decreased by the additive. It classically acts as a lubricant or separating agent that enables easy detachment, especially under shear conditions and higher stresses. In contrast, the flow additive Aerosil does not cause any of these effects. Neither in the case of low surface coverages, which were proven for the ground sample by SEM images (Figure 7D), nor for higher coverages, which are assumed for the raw sample due the constant concentration but lower specific surface area, can similar effects be observed.

**Figure 14:**
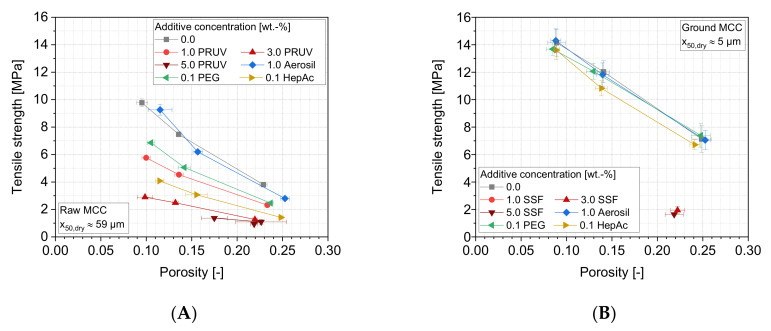
Tensile strength as a function of the tablet porosity for different additives: (**A**) raw MCC and (**B**) ground MCC. Due to capping, the tablets for ground MCC with SSF cannot be evaluated (except the tablets with 3% and 5% SSF, with a compaction stress of 100 MPa), *n* = 10.

In comparison to the lactose, MCC generally obtains higher tensile strengths (Figure 14). This can mainly be attributed to higher amounts of energy, which are plastically stored inside tablets of MCC, and the lower mean yield pressure, which is associated with ductile deformation behavior (Table 4). These points lead to an enhanced formation of contact areas between the particles while maintaining higher porosities, as compared with lactose. In the case of the raw MCC, comparable grinding aid effects as for lactose can be observed. However, a more pronounced decrease of tablet strength is caused by the liquid grinding aids, which emphasizes that the additives either act as material-specific in terms of their impacts on adhesion reduction or the effect of changes in specific surface area is determining. Regarding the latter, MCC will mostly maintain its specific particle surface area during compression as it is deforming plastically, but it will lose free surface area due to the generation of large inter-particle contacts. Lactose particles, on the contrary, break, and therefore, generate higher specific particle surface areas upon compression. Such new surfaces will not be covered with liquid grinding aids, which are believed to control the surface properties on the molecular level and are accordingly applied in lower concentrations than lubricants that act on a particulate level. Since the particle surface area of MCC only slightly changes during compression, a high surface fraction will be covered with the grinding aids also after compression and will facilitate a more pronounced weakening of the structure than for lactose. 

The compaction of ground MCC samples yields a different picture. Again, generally, higher tensile strengths were achieved due to the drastically increased specific surface area of the particles, and therefore higher contact density in tablets as compared with raw MCC samples, if intact tablets were yielded (Figure 7). Furthermore, no significant differences are seen for compacting samples with Aerosil, liquid grinding aids or no additives. Accordingly, it can be assumed that the coverage of the MCC surface is too low for these additives in order to cause a divergent effect regarding the adhesion forces between compacted particles or friction, especially as the specific surface area of MCC is approximately increased three-fold by grinding. When using SSF, in turn, the formation of sufficiently strong bonds between the MCC particles is mostly prevented, leading to a capping of the tablets during their ejection from the dye. It must be taken into account that jet milling processes not only facilitate comprehensive comminution, but also provide strong blending and dispersion performance, as also shown by the dispersion MCC particles themselves. Additionally, SSF particles are likely to be dispersed and distributed to the (new) MCC particle surfaces, yielding a high degree of coverage. In unity with the effect of high elastic fractions of the compression energies, which were seen for samples containing SSF, these findings explain why capping occurs for lubricants and low compaction stress (100 MPa), which also show low elastic energy fractions. Sufficiently stable tablets containing MCC and SSF are, however, of very high porosities and low strengths. 

**Figure 15:**
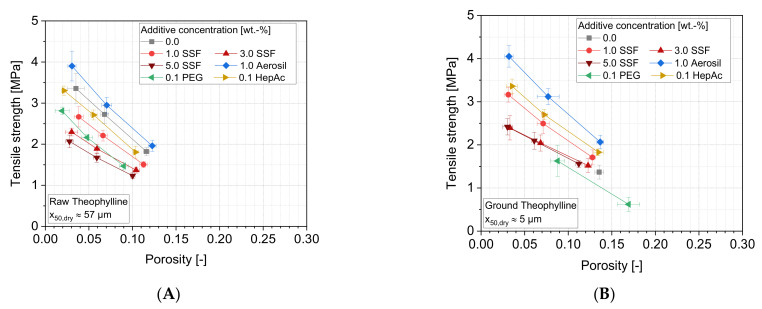
Tensile strength as a function of the tablet porosity for different additives: (**A**) raw theophylline and (**B**) ground theophylline. Due to capping, some tablets without additive and PEG cannot be evaluated, *n* = 10.

The compaction of theophylline leads to weaker tablets, in comparison to the other materials (Figure 15), even though comparatively low porosities are achieved. According to Table 4, significantly lower amounts of energy are necessary for the compression and are accordingly plastically stored inside the theophylline tablets, however, very large inter-particulate contact areas are expected because very low porosities were achieved. This is in accordance with its very low mean yield pressure, hinting at highly ductile deformation with low deformation resistance. Thereby, it is surprising that the micronization of the particles without any additive does not improve the tablet strength, but causes quite the contrary, e.g., reflected by an increased capping of the tablets. Accordingly, the strength of the particle contacts may be close to the strength of the material itself. Under such conditions and taking the low deformation resistance into account, the effect of particle sizes on aggregate strength is rendered unimportant. Since both the in-dye porosities and the stored energies do not differ significantly from the other samples, it can be assumed that the failure of the tablets is rather caused by high stresses inside the tablet while ejecting it from the dye. In addition, friction forces between the tablet and the dye may be higher in the case of non-stabilized, fine theophylline particles. However, no systematic abnormalities were seen for the corresponding ejection forces with the additive-free formulation, neither for the raw nor the ground samples (data not shown). It can therefore be assumed that it is more the internal stress conditions in the tablet that lead to mechanical failure than the friction between the tablet and wall. Which mechanisms finally lead to the failure cannot be clearly stated at this point and requires further investigations.

Additionally, mostly the expected additive effects can be seen for both the raw (A) and ground (B) theophylline samples. Interestingly, the liquid grinding aid PEG leads to higher porosities and much lower tablet strengths, especially in the case of the ground material, whereby partly, capping also occurs. In this case, the result corresponds to an uncommonly low powder flowability (compare Figure 9). However, again, no anomalies can be found regarding the in-dye porosities or the stored energies (data not shown). Therefore, it can be assumed that the low tablet strengths are caused by a weaker nature of the bonds at the contact areas rather than by the size and number of contact areas. It is also possible that liquid additives such as PEG can lead to a partial dissolution of the theophylline surface, making the bonds at the contact sites weaker. However, this cannot be proven at this point and can therefore only be regarded as a hypothesis.

### 3.5. Correlation of the Grinding and the Compaction Behavior

**Figure 16:**
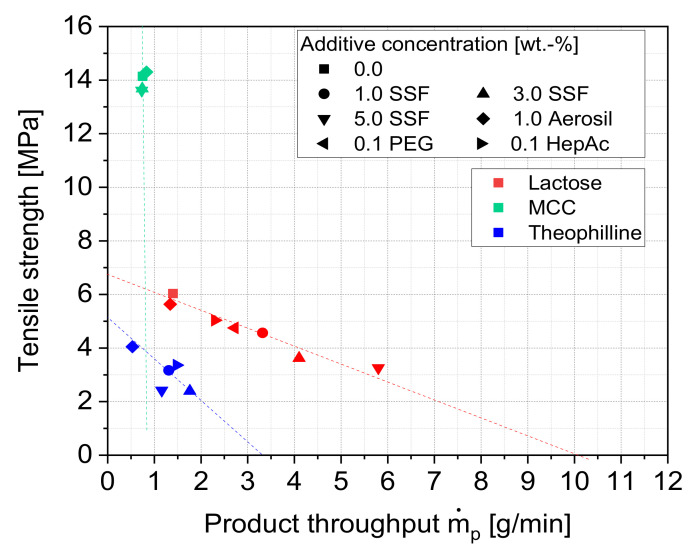
Correlation between the product throughput, $\dot{m_{p}}$, during the jet milling and the tensile strength of the ground and compacted powder samples (compaction stress = 400 MPa). Dashed lines act as a guide to the eye.

The results of this study emphasize that the investigated additives act by different, partly complex and solid-specific modes of action, leading to varying effects depending on the unit process. Regarding the grinding efficiency, positive additive effects are only achieved when the additive is capable of forming stable and nearly complete layers on the product particles. This is strictly necessary to avoid a direct contact of the particles, as the particles are usually colliding with high kinetic energies during jet milling. Consequently, the major benefit during jet milling can be attributed to a reduction of the adhesion forces between the particles, leading to a lower formation of strong agglomerates. This, however, also affects the strengths of the bonds within the produced tablets. In turn, it is favorable that either the particles themselves or the coating of molecules or additive particles break during compaction to display new, uncoated and, thereby, more bondable surfaces. Whether and to which extent this is happening depends on the interplay of the deformation behavior of the host particles and the surface interaction with and mechanical properties of the additives. More generally, Figure 16 demonstrates that the different additives cause opposing effects regarding the grinding and the compaction process. Especially lubricants and liquid grinding aids, which lead to an increase of the grinding efficiency by reducing the tendency of agglomeration, cause comparatively low tensile strengths of the produced tablets.

It can further be seen that the additives partly act as solid-specific, which can be attributed to different reasons. On the one hand, it can be assumed that the effectiveness of the additives is determined by the specific interactions between the additive molecules or additive particle surface and the solid particle surface, leading to differences regarding the particle–particle interactions or even the chemical stability of the particle surface. On the other hand, the deformation behavior and the microstructure of the particles have great influences on both the grinding as well as the compaction process. Additionally, the additives can influence process outcomes due to their deformation behavior, but probably to a lesser extent. It is shown that different additive classes such as lubricants or grinding aids may cause varying effects depending on these products’ properties. Especially the microstructure of the product material can suppress additive effects or overemphasize their effect under certain process conditions. This is also the reason why the correlation of product throughput and tensile strength displays logical trends for one material, but cannot be directly transferred from one material to the other (Figure 16). For instance, none of the additives affect the complex grinding mechanism of MCC particles during jet milling, yielding comparable throughputs. However, low tablet strengths or even tablet failures due to capping are observed in the case of lubricants. Furthermore, the effectiveness of the additives is strongly dependent on the coverage of the particle surface with additive molecules or particles. As the surface coverages vary for the investigated materials and additives, it is even more difficult to derive universal rules from the results that are shown. Further, the impacts of the additives on the resulting ground particles’ morphology and flowability are rather small, even though great effects are observed during both the grinding process and in the compaction products. Thus, it is not possible to predict the grinding or compaction behavior exclusively by the impacts of the additives on only one of these properties.

In general, the results of the work reveal great potentials of applying additives during the dry processing of organic materials. Especially the fine grinding can be improved in terms of efficiency and product quality. In the case of very adhesive and sticky materials, the dry micronization would even be impossible without the addition of such additives. However, this inverse correlation, where the positive effects in grinding are correlated with negative effects in compaction, emphasizes that the additive application can also be restricted by subsequent process steps, such as the tableting of the ground material. Depending on the product material and the required product properties, an optimum must be identified towards the process throughput.

## 4. Conclusions

Within this study, three different classes of additives—particulate lubricant, nano-disperse flow additive and liquid grinding aids—were investigated in terms of their impacts on the dry processing of organic model materials with different deformation behavior as well as their influences on the corresponding powder properties. The additives caused varying and mostly opposing effects during the dry fine grinding process and the compaction of the materials. Thereby, especially liquid grinding aids and lubricants were shown to reduce the agglomeration behavior of the particles, and thereby, to improve the grinding efficiency. For certain products such as theophylline, stable and efficient grinding conditions were impossible without the addition of an additive. The positive effects of an additive are characterized by a reduction of the adhesive forces between the particles. Particularly, well-performing additives in the fine grinding process led to disadvantageous effects during the tableting of the material. For instance, much lower tablet strengths were achieved for additives such as grinding aids and lubricants. The flow additive Aerosil, in turn, did not improve the grinding efficiency, but did not decrease the tablet strengths either.

Furthermore, the impacts of these additives on the particle and powder properties are rather small, at least in the investigated concentration range. For instance, only a slight reduction of the surface energy was observed, unlike what is commonly found for inorganic materials. Additionally, the microstructure of the product material may be much more versatile and critical in the case of organic materials. The dispersion of filamentous particle structures during the micronization of MCC and the associated high specific surface area led to completely different additive effects than those seen for materials such as lactose and theophylline. The complex interaction between the type of additive, the microstructure and the material deformation behavior of the product, as well as the mechanical (stress) conditions during the processing, makes it highly complex to derive universal rules for the application of such additives.

Overall, the results showed great potentials for additive applications in dry processing of organic materials, especially in terms of increasing the grinding efficiency as well as the product fineness or avoiding product adhesions on the machine equipment. At the same time, certain limitations of the additive application and the need for a balancing of their application were revealed, as additives also cause negative impacts on subsequent process steps, such as the tableting of the ground material. The extent and relation of the effects in different processes mostly rely on deformation and interaction behavior, however, due to their complexity, they require further investigation to broaden and generalize knowledge as well as derive transferrable models.

## Figures and Tables

**Table 1:** Physical and chemical properties (based on own experimental results).

	Lactose	Microcrystalline Cellulose (MCC)	Theophylline
Solid density (g/mL)	1.535	1.562	1.445
Bulk density (g/mL)	0.587	0.371	0.406
Median particle size x_50,dry_ (µm)	113.6	59.6	57.2

**Table 2:** Additives with manufacturer data.

Substance Class	Name	Manufacturer	Physical State	Boiling Point (°C)	BET-Surface (m^2^/g)
Carboxylic acid	Heptanoic acid 96% (HepAc)	Sigma Aldrich	liquid	223	-
Poly glycol	Polyethylene glycol 200 (PEG)	Sigma Aldrich	liquid	>150	-
Flow additive	Silicon dioxide (Aerosil 200)	Evonik	solid	-	175–225
Lubricant	Sodium stearyl fumarate (SSF)	JRS Pharma	solid	-	1.6

**Table 3:** Powder flow behavior according to Jenike [43].

$ff_{c}$	Powder Flow Behavior
$ff_{c}$ < 2	Very cohesive and not flowing
2 < $ff_{c}$ < 4	Cohesive
4 < $ff_{c}$< 10	Easy flowing
10 < $ff_{c}$	Free flowing

**Table 4:** Elastically and plastically stored energy during the compaction of 300 mg of materials ground without any additives at stresses of 400 MPa.

Material	Elastically Stored Energy (J)	Plastically Stored Energy (J)	Elastic/Plastic Ratio φ (−)	Mean Yield Pressure (MYP) (MPa)
Lactose	0.5	11.8	0.04	150
MCC	1.1	15.6	0.07	105
Theophylline	1.4	7.6	0.19	70
